# Troubleshooting a Rare Anatomic Variation with Intraoperative Navigation in a Patient with Bilateral C2 Pars Fractures

**DOI:** 10.7759/cureus.4427

**Published:** 2019-04-10

**Authors:** William Clifton, Christopher Louie, Eric Nottmeier, Mark Pichelmann, Selby G Chen

**Affiliations:** 1 Neurosurgery, Mayo Clinic, Jacksonville, USA; 2 Neurosurgery, Mayo Clinic, Jacksonville , USA; 3 Neurosurgery, Mayo Clinic, Rochester, USA

**Keywords:** c2, pars, atlas, hangman's fracture, navigation, spine, instrumentation

## Abstract

C2 pars fractures occur most commonly after traumatic hyperextension injuries. Although a significant number of cases may heal with conservative measures, some require surgical intervention. Anatomical variations of the V3 segment of the vertebral artery are not uncommon and may present an obstacle to safe instrumentation. Intraoperative CT-guided navigation is a useful tool in these cases, but the limitations of accuracy in the upper cervical spine especially in the context of unstable injuries must be understood to avoid complication. In this case we present a rare anatomic variation of the vertebral artery size and position in conjunction with bilateral C2 pars fractures treated successfully by surgical fixation. This article highlights the important technical details of the posterior instrumentation of unstable atlas pars fractures with the aid of intraoperative navigation.

## Introduction

Pars fractures of the second cervical vertebrae occur most commonly after traumatic hyperextension injuries [1]. The majority of these rare fractures may heal with external orthosis; however, fractures with significant displacement or angulation require surgical intervention [1-3]. The vertebral artery is a structure in close proximity to the target area for screw placement, and careful preoperative assessment of its position in the vertebral foramen as well as the dorsal extent over the arch of C1 is the key to avoiding injury during atlantoaxial fixation. Anatomical variations in the V3 segment of the vertebral artery are not uncommon and may present an obstacle to safe instrumentation. Intraoperative CT-guided navigation is a useful tool in these cases, but the limitations of accuracy in the upper cervical spine must be understood to avoid complication. In this case we present a rare anatomic variation of the vertebral artery size and position unilaterally in conjunction with bilateral pars fractures treated successfully by a posterior C1-3 fusion with unilateral C2 pedicle fixation using intraoperative navigation.

## Case presentation

**Figure 1:**
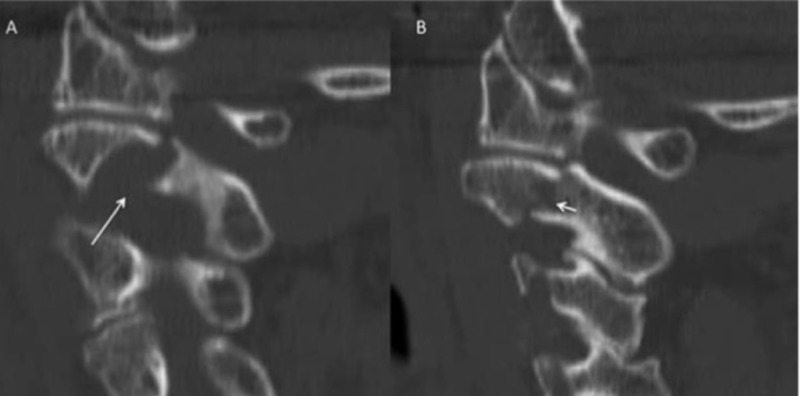
Sagittal noncontrasted CT scan. (A) Large vertebral foramen at C2-3 (long arrow) with a fracture superiorly in the C2 pars on the right side. (B) Displaced fracture through the left C2 pars and pedicle (short arrow).

**Figure 2:**
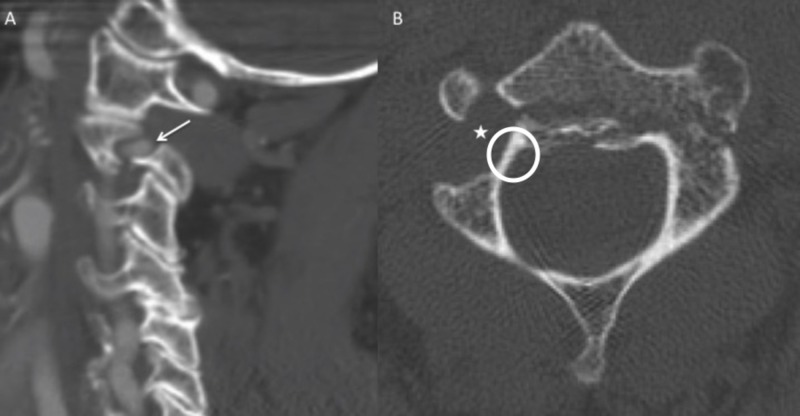
Computed tomographic angiogram (CTA) images. (A) Sagittal CTA images showing the large right dominant vertebral artery with congenital dehiscence of the superior portion of the foramen (arrow). This was an important consideration in exposure of the right C1 lateral mass to avoid iatrogenic vertebral artery injury. (B)  Noncontrasted axial CT showing a large vertebral foramen on the right side (star) with a congenitally small C2 pedicle (circle). A horizontal fracture through the posterior portion of the vertebral body is also seen.

A 78-year-old woman was involved in a motor vehicle collision while traveling approximately six weeks before presenting to our institution. She initially had high cervical neck pain at the time of the event but no other neurologic symptoms. She was brought to a local trauma center at the time of the event, and a computed tomographic angiography (CTA) of the neck revealed a Levine and Edwards Type II fracture with bilateral C2 pars and pedicle fractures extending into the vertebral body with anterolisthesis of C2 on C3 (see Figure 1). Also seen was a tortuous right dominant vertebral artery that filled a large C2 transverse foramen with a congenitally small pedicle (see Figure 2). Her vertebral artery on the left appeared to contribute very little to her posterior circulation. There was no evidence of radiographic vascular injury. She was advised to undergo surgical fixation at the time of her injury, however, she elected to wait until she returned home. She was discharged from the outside hospital with a hard cervical collar and presented to our institution for further evaluation over a month later. After discussing the possible treatment options, including continued conservative treatment with continued external orthosis vs. surgical intervention, the patient elected for surgical intervention. The risks and benefits of the surgical options were discussed with her in detail, including an anterior approach at C2-3, or a posterior C1-3 fusion. The patient elected to have a posterior fusion to avoid the possible swallowing complications of a high cervical exposure and other possible risks of an anterior approach..

**Figure 3:**
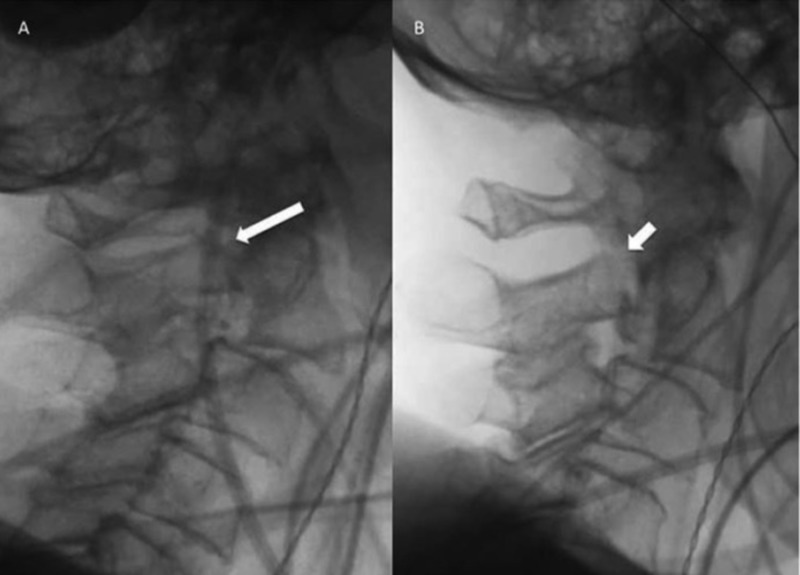
Intraoperative lateral fluoroscopy. (A) Pre-positioned displaced fracture (long arrow) through the C2 pars and pedicle with anterior fish mouthing of the disc space and anterolisthesis of C2-3. (B) Post-positioning and manual reduction of the fracture with improvement in left pedicle alignment (short arrow) as well as the C2-3 anterolisthesis.

**Figure 4:**
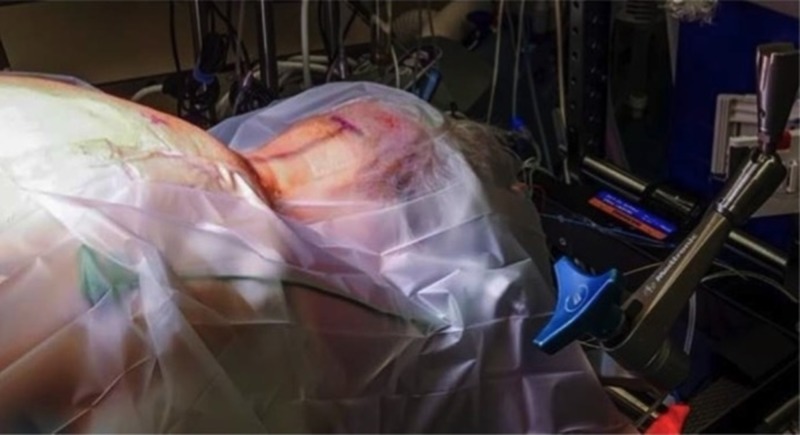
Patient in prone position with attachment of the cranial reference arc for intraoperative navigation.

Informed consent was obtained and the patient was brought to the operating room. Neurophysiologic monitoring was utilized to establish baseline motor and somatosensory evoked potentials. After application of cranial pinions, the patient’s neck was brought into a neutral and slightly flexed position under live fluoroscopy. A post-positioning film showed the patient’s anterolisthesis had reduced and the fractured pedicle showed improved alignment (see Figure 3). Motor-evoked potentials and somatosensory-evoked potentials showed no change from baseline. A cranial reference frame was attached to the head clamp for CT-guided intraoperative navigation (see Figure 4). Attachment of the cranial reference frame to the head clamp was a key step to ensure maximal accuracy of the optical system during instrumentation, as the posterior elements of C2 would not be stable enough to accept the spinous process clamp after exposure.

**Figure 5:**
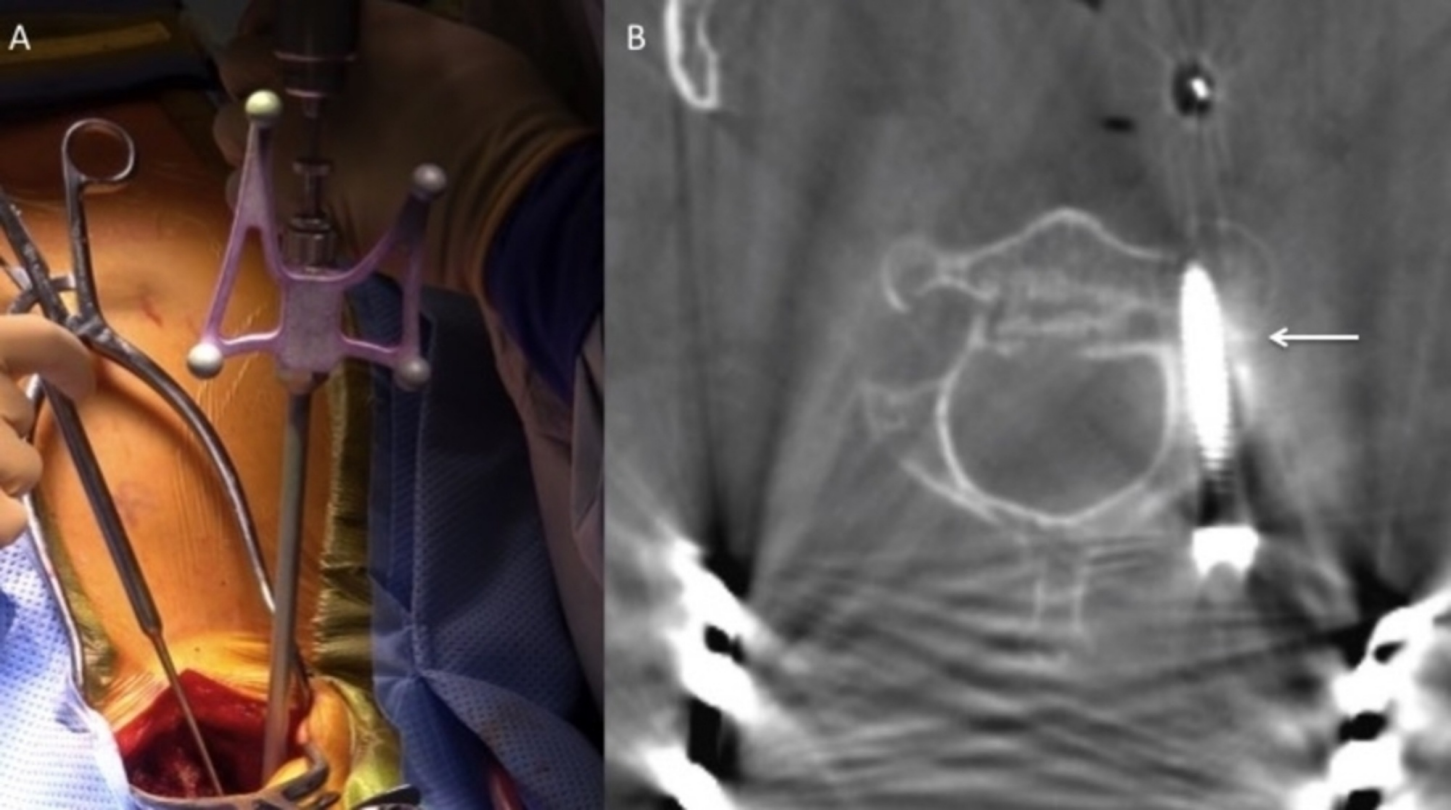
Intraoperative left C2 pedicle screw placement across the fracture line using navigation. (A) Intraoperative photo showing use of a navigated drill in order to access the left C2 pedicle across the fractured segment. (B) Post-instrumentation intraoperative imaging showing left pedicle screw placement across the fracture line (arrow) and into the C2 vertebral body with good purchase and alignment.

**Figure 6:**
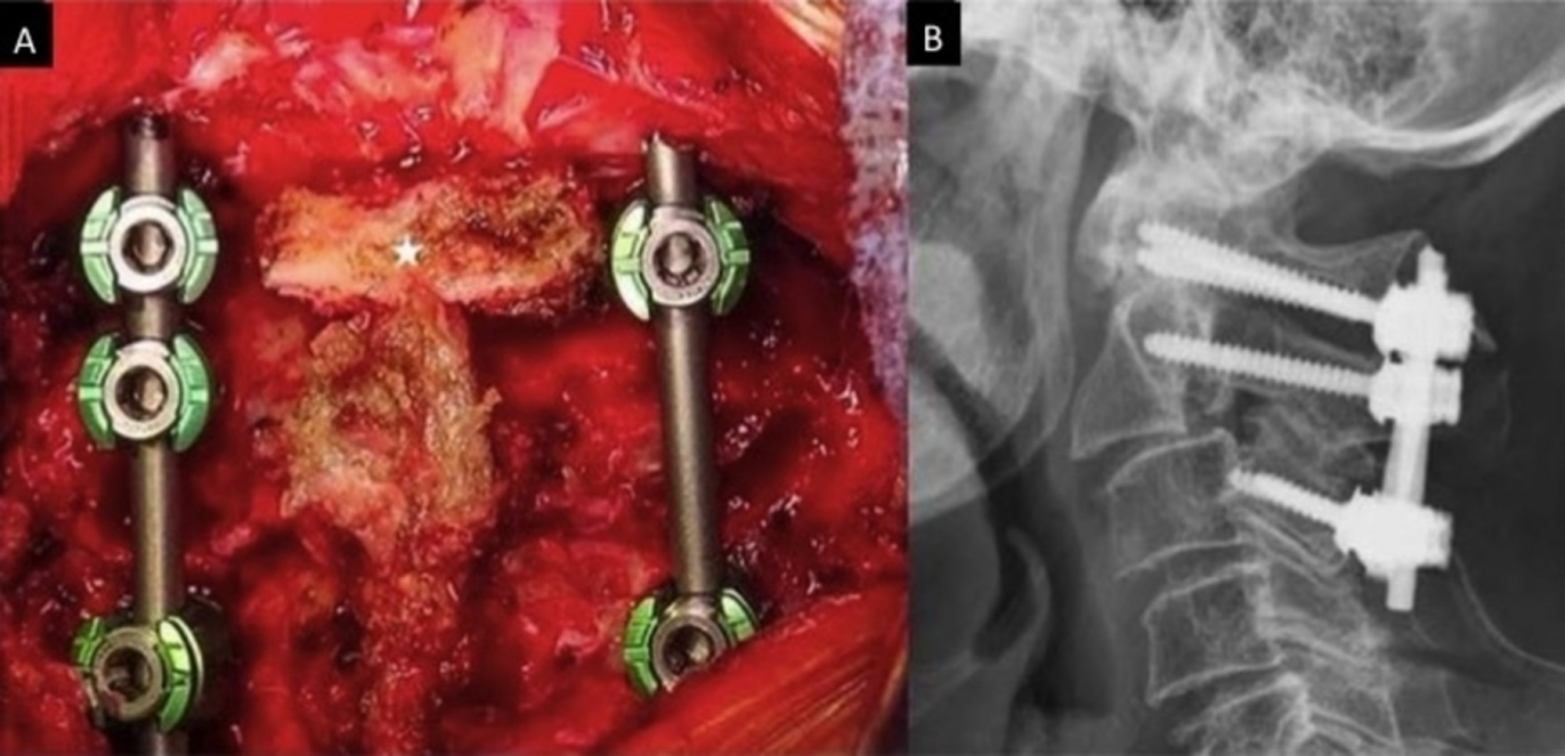
Final construct and postoperative X-ray. (A) Intraoperative photograph of the final C1-3 construct. A small piece of iliac crest graft (star) was placed in between the decorticated C2 and C1 laminae for fusion material. (B) Postoperative lateral X-ray showing stability of the construct post-mobilization.

After exposure of the posterior elements from C1-3 and the C1 lateral masses, an O-arm spin was performed for navigation registration. The left C2 pedicle was accessed using a navigated drill. The fracture line in the middle of the pedicle was crossed with the drill bit into the vertebral body. Careful palpation of the drill track with a ball-tipped probe did not show any breaches. The hole was tapped with a navigated 3.5-mm tapered tap. A 4.0 mm x 24 mm screw was slowly advanced across the fracture into the C2 vertebral body. Care was taken during screw placement to ensure purchase across the fracture so that the tip of the pedicle screw did not push the vertebral body anteriorly. Bilateral C1 and C3 lateral mass screws were then placed. The right C2 pedicle was skipped due to her congenital abnormality and the risk of vertebral artery injury. An intraoperative CT scan showed excellent hardware placement (see Figure 5). A cortico-cancellous piece of iliac crest was harvested and contoured to an appropriate dimension for placement between the C1 and C2 lamina. The laminae were decorticated and a piece of iliac crest graft was placed in between. Bilateral titanium rods were cut and placed. The wound was closed in layers and the patient awoke with no complications. She was placed in a hard cervical collar post-operatively. Postoperative X-ray images were obtained after the patient mobilized and showed stability of the construct (see Figure 6).

## Discussion

C2 pars fractures that have significant displacement or discoligamentous injury often require surgical fixation [3]. There have been many techniques described in the literature, depending on the extent and biomechanics of the injury [3-8]. Intraoperative navigation has provided a significant benefit in cases of fractures with distorted anatomy and has been shown to reduce intraoperative radiation exposure [9-10]. Use of the cranial reference arc is the key in trauma cases when spinous process fixation is not possible [11-12]. Anatomic variations in the position of the vertebral artery at the craniocervical junction are not uncommon [13-14]. It is important to localize the position of the artery before surgical intervention to avoid iatrogenic injury. In our patient’s case, her large right dominant vertebral artery was the main supplier of her posterior circulation, and, therefore, was vital to preserve. Her right pedicle had thinly remodeled in conjunction with a large loop of artery at the C2 transverse foramen, which likely made the bone susceptible to fracture. 

This case presented a unique challenge for atlas fixation. The anatomic variation of the right vertebral artery did not allow for screw placement on this side. Fixation to the posterior elements through a right C2 laminar screw or short pars screw would not be biomechanically sufficient due to the complete fracture through the small pedicle. The primary fixation point of the construct that would ensure fracture stability was through the left C2 pedicle into the C2 vertebral body through the fracture line. Preoperative manual reduction of the fracture allowed for better alignment of the fractured left pedicle and a straight target for pedicle screw insertion across the fracture line. During drilling, tapping, and screw placement across the fracture it was important to maintain continuous awareness of tactile feedback to ensure purchase across the fracture line and to avoid pushing the distal part of the fracture anteriorly. Iatrogenic displacement of the fractured pars would not have been exhibited in real time through the navigational system, and it is important to keep this concept in mind during instrumentation of unstable spinal fractures when using navigation, as the optical system may lose accuracy if the fractured segments change position throughout the case. Interpretation of tactile feedback from direct palpation as well as direct visualization of the spinal elements during instrumentation is essential to avoid errors in navigation and misplacement of hardware.

## Conclusions

Anatomic variations of the vertebral artery position at the C1-2 level are not uncommon. This case presented a rare challenge for fracture stabilization due to the position of the dominant right vertebral artery as well as the patient’s congenitally small right C2 pedicle. With the aid of intraoperative navigation and careful preoperative radiographic assessment, adequate spinal fixation through the left pedicle fracture line was achieved and iatrogenic vertebral artery injury was avoided. This case highlights the important technical details of the posterior surgical treatment of unstable atlas pars fractures with the aid of intraoperative navigation.
